# Long lived second mode internal solitary waves in the Andaman Sea

**DOI:** 10.1038/s41598-020-66335-9

**Published:** 2020-06-24

**Authors:** J. M. Magalhaes, J. C. B. da Silva, M. C. Buijsman

**Affiliations:** 1 Department of Geoscience, Environment and Spatial Planning (DGAOT), Faculty of Sciences University of Porto, Porto, Portugal; 2 Interdisciplinary Centre of Marine and Environmental Research (CIIMAR), Matosinhos, Portugal; 3 Division of Marine Science, University of Southern Mississippi, Stennis Space Center, MS, USA

**Keywords:** Ocean sciences, Physics

## Abstract

Internal waves are density oscillations propagating along the ocean’s inner stratification, which are now acknowledged as a key constituent of the ocean’s dynamics. They usually result from barotropic tides, which flow over bottom topography, causing density oscillations to propagate along the pycnocline with a tidal frequency (i.e. internal tides). These large-scale waves propagate away from their forcing bathymetry and frequently disintegrate into nonlinear short-scale (higher-frequency) internal wave packets. Typically, short-scale internal wave observations in the ocean are associated with vertical structures (in the water column) of the lowest fundamental mode. Higher vertical modes have recently been documented as well, but these are commonly short-lived (up to a few hours). However, unprecedented satellite images showing long-lived short-scale mode-2 internal waves have now been documented in the Andaman Sea, and we report here the companion results of a non-hydrostatic and fully nonlinear numerical model. The simulations reproduce the waves’ main characteristics as observed in satellite imagery, and the results suggest a resonant coupling with a larger-scale mode-4 internal tide as an explanation for their long-lived character.

## Introduction

Ocean sciences are increasingly relying on satellite remote sensing. In particular, our understanding of Internal Waves (IWs, i.e. density oscillations propagating along the ocean’s inner stratification) has seen considerable advances in the last decades owing to satellite imagery^1–3^. For instance, IW fields obtained via satellite images are widely used in planning and performing *in situ* measurements^4,5^, validating numerical models^6,7^, and bridging IWs with other fields of ocean sciences^8,9^.

The Andaman Sea in the Indian Ocean is a classical study region for IWs dating back to the 19^th^ century^10^, and staged some of the first dedicated IW measurements in the open ocean^11^. This region is in fact unique, in the sense that a series of IW generation hotspots occur along a meridional 1000 km island ridge, each with its own distinct characteristics concerning bathymetry, currents and stratification. According to Osborne and Burch^11^, very large-scale IWs are tidally generated along the ridges of the Andaman and Nicobar Islands which then propagate eastwards while retaining their shape and speed for considerable distances. We note that these waves have typically vertical structures of the fundamental mode (i.e. mode-1), and may be predicted with the shallow water Korteweg and deVries equation, and hence the term Internal Solitary Waves (ISWs) is usually referred to when describing the IWs in the Andaman Sea.

**Figure 1:**
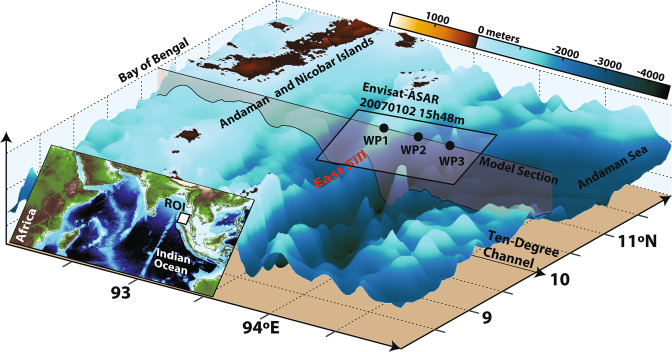
Study region in the western Andaman Sea of the Indian Ocean. The region of interest (ROI) is also shown in the inset in the left corner. The Envisat-ASAR acquisition discussed in Fig. 2 is outlined by a black rectangle for reference, along with the model section used in this study (see grey shaded section). See text for more details. Bathymetry data from Smith and Sandwell (1997) were used in this figure, and processed using Matlab (R2019a, https://www.mathworks.com/products/new_products/release2016a.html) and Adobe Illustrator (CS5, https://helpx.adobe.com/illustrator/illustrator-cs5-cs55-tutorials.html).

Several studies have emerged since Osborn and Burch^11^ – all of which revealing powerful ISWs in this region which are nonetheless typically mode-1 in their vertical structure^12^. However, Synthetic Aperture Radars (SARs) have been recently used^12,13^ in documenting the existence of long-lived mode-2 solitary-like waves propagating eastwards along the Ten-Degree Channel of the Andaman Sea (see Fig. 1 for locations). Note that, ISWs are particularly well observed in SAR images, essentially owing to their short-scale modulations of sea surface roughness, which in turn result from the waves’ surface current gradients^14,15^. Furthermore, SARs have particularly high spatial resolutions and can distinguish different IWs vertical modes. In fact, the mode-2 observations reported in the Andaman Sea consistently present a dark reduced backscatter preceding a bright enhanced backscatter in the propagation direction of the wave (for more details see Magalhaes and da Silva^12^, and references therein).

**Figure 2:**
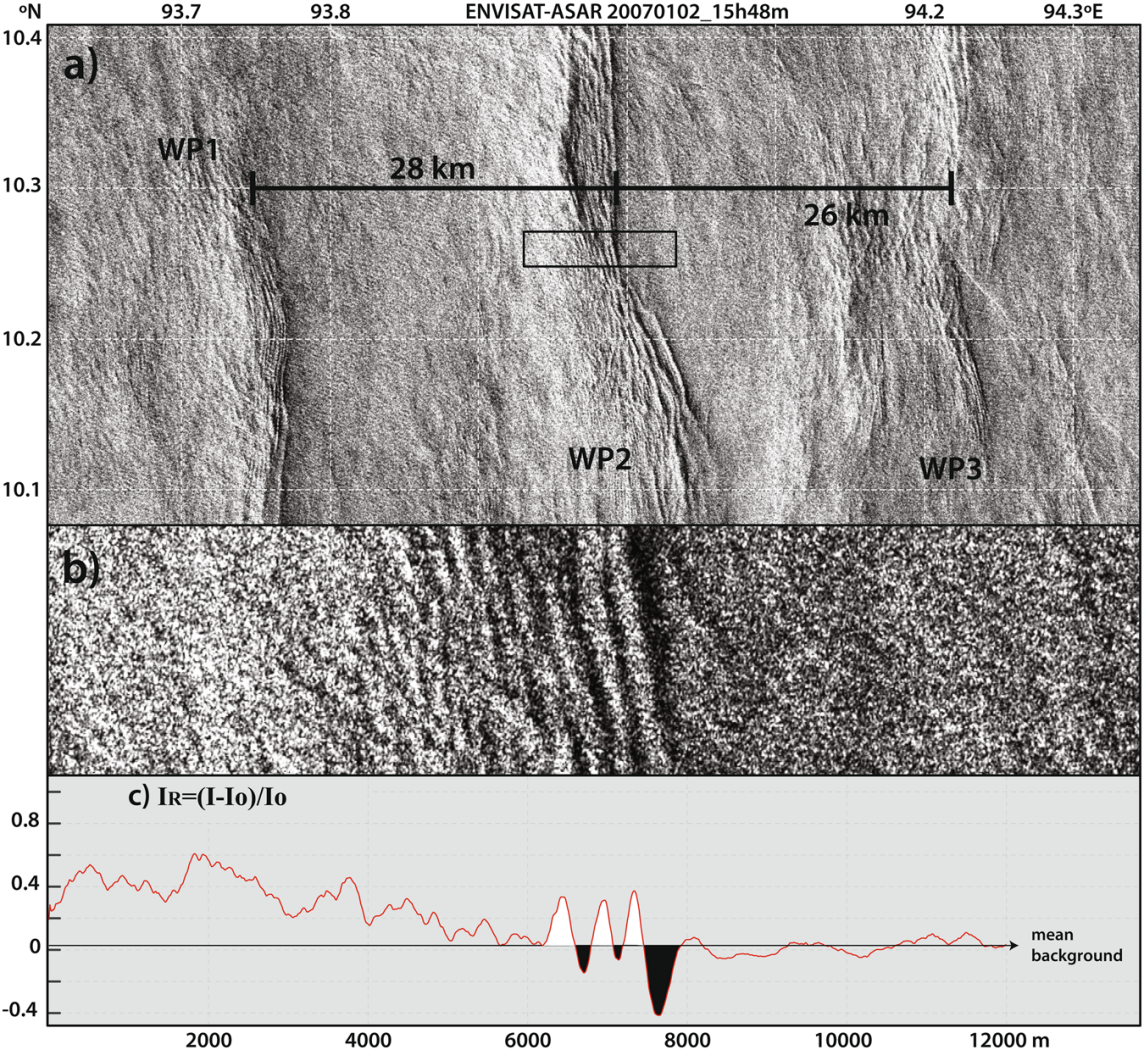
(**a**) Subset of an Envisat-ASAR acquisition dated January 2^nd^ 2007 at 15h48m. The image shows three wave packets of mode-2 solitary-like waves propagating eastwards along the ten-degree channel in the Andaman Sea (labelled WP1 to WP3). (**b**) Zoomed section corresponding to black rectangle in top panel showing that a leading darker band precedes each wave in their direction of propagation. (**c**) Transect corresponding to middle panel given in relative intensities (I_R_, where I are intensities from the SAR image and Io is a measure of the unperturbed background). Note that negative/positive sections of the waves are filled in black/white for reference.

An example of these waves is shown in Fig. 2. Interestingly, similar examples have been documented in the literature only on few occasions, but always exhibiting short-lived characteristics (typically a few hours^6,16^). In the Andaman Sea, however, they can persist for more than one day, and hence (by comparison) are considered hereafter as being long-lived in this study region. This is seen in Fig. 2, where three packets of waves are observed, and hence assuming a packet is generated every 12.42 hours, propagation can be hypothesized during three semidiurnal tidal cycles.

The dynamics of higher-mode solitary-like waves is an active field of research. For instance, seasonal observations of mode-2 ISWs in Yang *et al*.^17^ have been shown to be strongly correlated with optimum winter stratification in the South china Sea – with typical thermoclines around 150 m, and two well mixed layers above and below it. Particularly interesting to this study, is the numerical modelling presented by Zhang *et al*.^18^. According to these authors, background shear may cause shear-induced waves to radiate away from mode-2 ISWs (see e.g. their Fig. 4), which means additional mixing and even overturning in the case of strong shear. In fact, while modelling the shoaling process of mode-2 ISWs observed off the New Jersey coast, these authors have found that waves become highly dissipative, lasting only for about 5 hours (and 5 km) as observed by Shroyer *et al*.^16^.

While stratification and shear help clarify (at least to some extent) the short-lived character of mode-2 waves documented in the literature, some other mechanisms must be at work to explain their exceptionally large propagation paths observed in SAR in the Andaman Sea (cf. Fig. 2). Energy exchange via resonance between two oscillating systems is a common phenomenon in the natural world, which could explain these waves extended longevity. It is widely documented in natural sciences^19^, including in physical oceanography and in the framework of IWs in the ocean. Particularly interesting to this study are the numerous works describing resonant interactions between short and long waves in nonlinear dispersive wave systems^20^, including different vertical modes^21^, and between internal and surface waves propagating in stratified flows^22–24^ – as is the case of the waves observed in the Andaman Sea.

Therefore, a resonant energy exchange was hypothesized to be at work between a long (hydrostatic) mode-4 internal tide (i.e. an IW of tidal period) and the longed-lived mode-2 solitary-like IWs observed in the SAR^12^. Nonetheless, this previous work relied on SAR and linear theory to infer the waves’ vertical structure, which can only convey the ocean’s two-dimensional surface properties (i.e. the waves’ vertical structure needs either *in situ* sampling or numerical modelling). To confirm or dismiss this hypothesis we now turn to numerical modelling, given that dedicated *in situ* data is lacking in this remote region of the world’s ocean. This study is therefore designed to investigate whether or not a resonance coupling between short mode-2 solitary-like waves and a large-scale mode-4 IW is relevant in the Ten-Degree Channel of the Andaman Sea.

## Results and discussions

### Model validation via SAR imagery

Validating model results in this case is done in the same fashion as da Silva *et al*.^6^ (for ISWs in the Mascarene Ridge of the Indian Ocean). This means that simulations are considered to render the waves’ vertical structure correctly if they reproduce the essence of their two-dimensional horizontal structure as observed in SAR images.

**Figure 3:**
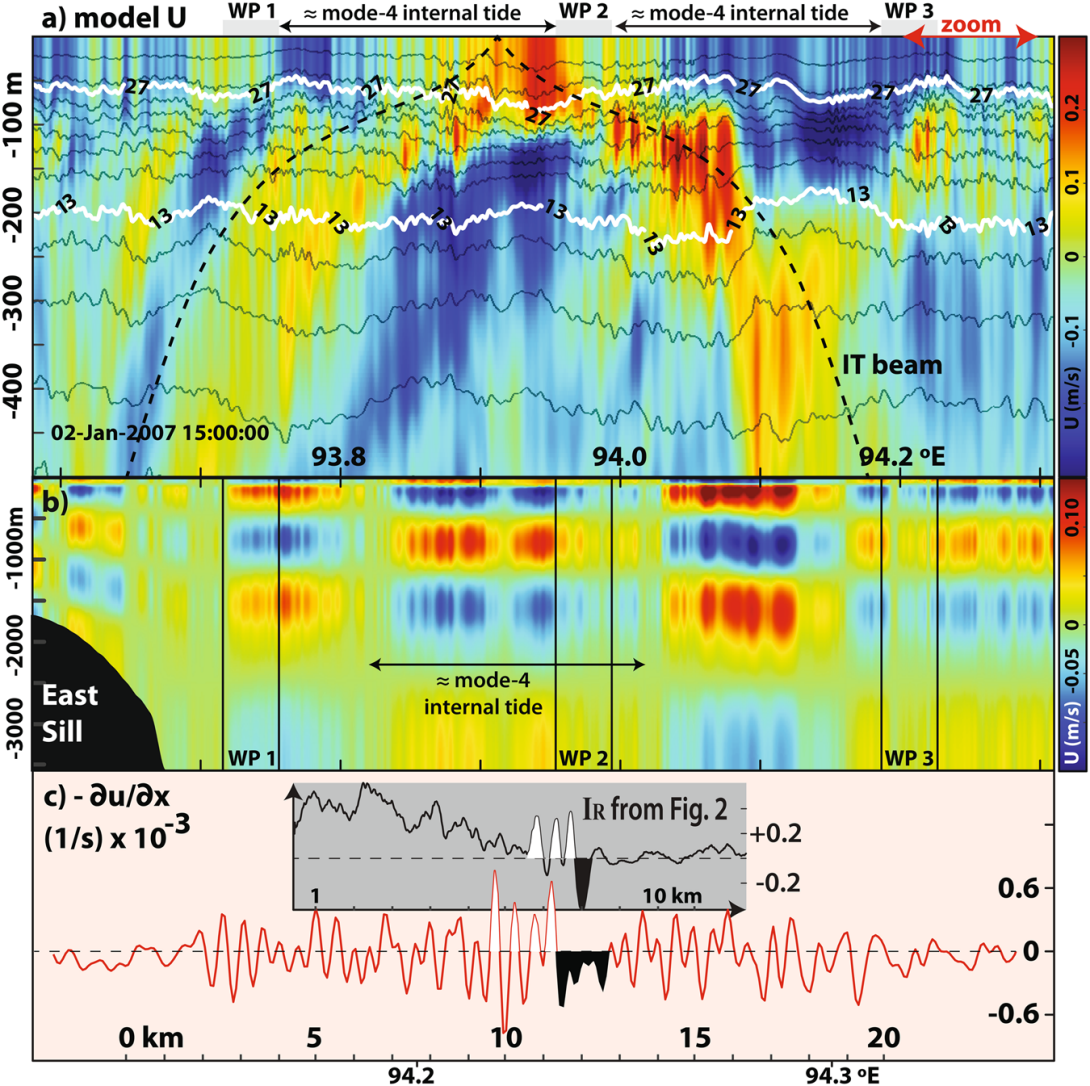
(**a**) Vertical section showing modelled horizontal velocities (*U*, colour coded in m/s on the right-hand side) for a time frame close to the SAR acquisition shown in Fig. 2. Selected isopycnals are overlaid for reference together with an internal tidal beam (dashed black line) computed from the model background stratification as in da Silva *et al*.^6^. Note that the approximate locations of the wave packets observed in the SAR are indicated with grey rectangles (WP1 to WP3). (**b**) Mode-4 horizontal velocities extracted from top panel using a modal decomposition, showing a long-linear mode-4 internal tide. (**c**) Modelled surface velocity gradients along WP3 (spanning the red arrow in top panel). Data from Fig. 2c is shown in an inset for comparison.

A series of comparisons between the waves observed in the SAR and those in the numerical simulations are shown in Fig. 3, which use a representative time close to the SAR observations shown in Fig. 2 – i.e. model time corresponds to January 2^nd^ 2007 at 15 h UTC (48 minutes before the SAR). In Fig. 3a, a zoom-in is shown with the modelled horizontal velocity field (*U*) and some selected isothermals. For comparison, the approximate locations of the waves observed in the SAR are given by the vertical grey rectangles (labelled from WP1 to WP3 as in Figs. 1 and 2), whose widths are about 5 km and hence representative of a typical width in a wave packet. It is interesting to note that these results show no evidence of the usual mode-1 ISWs that have been modelled in other known ISW hotspots as the Mascarene Ridge^6^ or the South China Sea^25,26^. In fact, no mode-1 ISWs were ever observed over the course of our simulations (i.e. along the Ten-Degree Channel). We recall this is consistent with the satellite imagery, since mode-1 waves are not observed here as well^12^. However, a closer inspection reveals high-frequency wave-like structures centred at the thermocline (i.e. around 150 meters deep), which are consistent with mode-2 solitary-like waves. These are especially well seen at WP3 as the isothermals wiggle in bulge-like features in the upward and downward directions (see isothermals between thick white lines). We note, that similar features have been identified in other studies as being mode-2 solitary-like waves using *in situ* measurements^16,27,28^ and in numerical models^18^. In the particular case of WP3, the corresponding wave features seen in the model are about 10 meters in amplitude and align fairly well (in their direction of propagation) with the SAR observations. Similar features may be seen as well close to WP1 and WP2, which are nonetheless somewhat smaller and harder to identify against the background flow, owing mainly to internal tidal beams (see dashed black line in Fig. 3a) interacting with the thermocline (near WP1) and undergoing surface reflections (near WP2).

The inter-packet distances observed in the SAR along this particular stretch of the Andaman Sea are roughly 30 km, and hypothesised to match the wavelength of mode-4 internal tides^12^. To investigate this hypothesis we may separate the modelled velocity field in Fig. 3a in its vertical modes and examine the existence of a long mode-4 internal tide. This procedure is usually done using a modal decomposition, which essentially solves a Sturm-Liouville boundary value problem governing linear IW motion in a stratified flow (see details in the Methods Section).

The isolated contribution for a mode-4 is shown in Fig. 3b. According to this result the modelled mode-2 ISW-like waves are travelling on a background structure consistent with a mode-4 internal tide with wavelengths of about 23 km. In this particular time frame the magnitude of the mode-4 contributions increases downstream of the forcing bathymetry (near 93.6 °E) and then decreases after WP3 – meaning it is stronger roughly between the SAR observations. Note also that, according to linear theory the horizontal velocities in a mode-4 vertical structure should change signs five times in the vertical^29^, which is in fact the case between the model’s surface and bottom boundaries. Therefore, the model is consistent with a mode-4 internal tide propagating across the domain, whose wavelengths agree well with the mode-2 ISW-like inter-packet separations seen in the SAR (cf. WP1 to WP3). In fact, the inter-packet distances in the SAR are slightly larger, which is usually interpreted as a nonlinear effect associated with solitary-like waves, which cause phase speeds to increase by about 20% when comparing with linear waves.

To further investigate the consistency between the SAR and the model it is possible to assess – at least qualitatively – if the mode-2 ISW-like features in Fig. 3a could leave a measurable surface signature in the SAR. This is done in same fashion as da Silva *et al*.^6^ and essentially means that −∂u/∂x may be used as a proxy for ISW signatures in SAR with brighter and darker areas (in comparison with an unperturbed background) corresponding to surface convergence and divergence, respectively^6,14^. This is shown in Fig. 3c, and according to these results the waves in the model induce surface velocity gradients of the same order as those in da Silva *et al*.^6^ (see their Fig. 8). As expected, they are smaller when compared with the larger and stronger mode-1 ISWs in the Mascarene Ridge. Interestingly, however, they appear similar in magnitude to the smaller-scale wave-tails, which also result from resonance with larger-scale IWs.

Finally, the higher frequency patterns seen in the SAR corresponding to mode-2 waves also appear to be in the modelled −∂u/∂x (see Fig. 3c and compare with the inset taken from Fig. 2). The waves’ leading sections with decreased backscatter seen in the SAR appear in Fig. 3c to correspond approximately to a negative section of −∂u/∂x, while increased backscatter compares roughly well with an increase in -∂u/∂x. Note that, these comparisons are made using the model’s raw surface velocities (i.e. unsmooth data) and hence additional background noise is expected to envelope these features.

### Evidence for resonant coupling between short mode-2 solitary-like waves and a long mode-4 internal tide

Altogether, Fig. 3 suggests the vertical structure in the model is consistent with the waves’ two-dimensional horizontal structure seen in the SAR. In particular, it reproduces the short-scale mode-2 solitary-like waves as well as their inter-packet distances (around 30 km). Therefore, we now turn to the model vertical structure to investigate a resonance condition between the mode-2 solitary-like waves and a longer mode-4 internal tide^12^. Note that, while mode-2 solitary-like waves are easily seen in the SAR, it is the model that provides the underlying vertical structure to confirm or dismiss the existence of a large-scale mode-4 internal structure.

**Figure 4:**
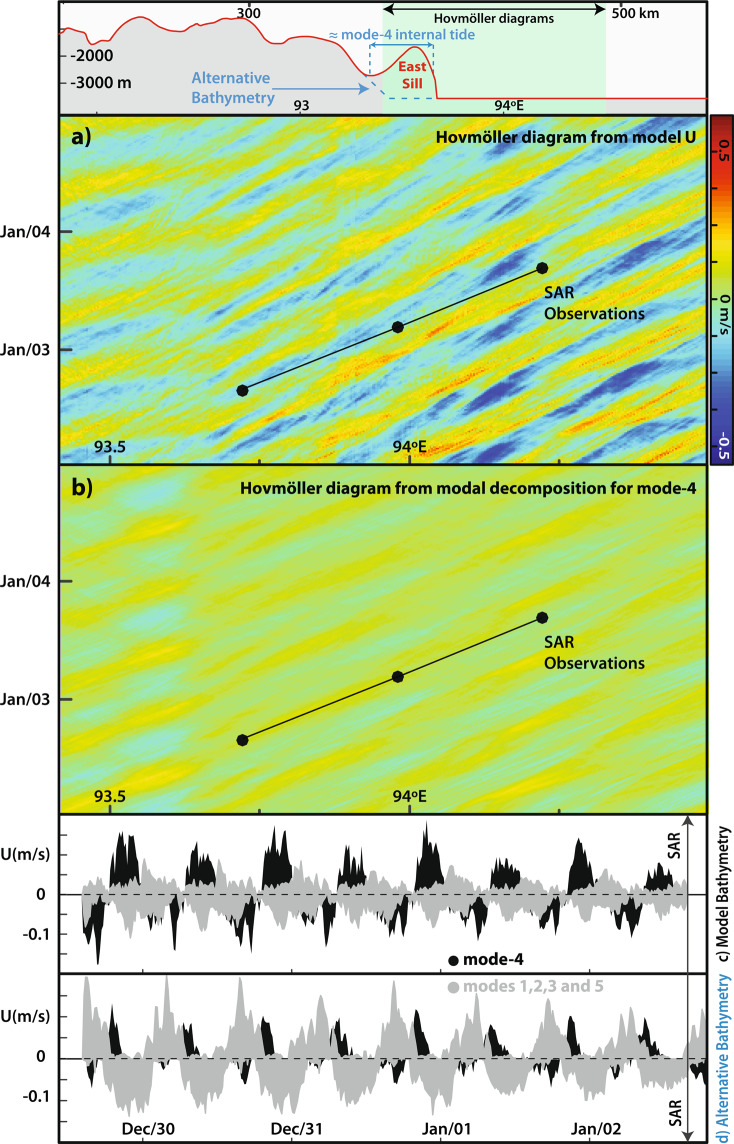
(**a**) Hovmöller diagram using horizontal velocities from the model along the thermocline (i.e. z = −105 m). The corresponding model bathymetry section is also shown at the top for reference (highlighted in light green). (**b**) Same as top panel using a modal decomposition for a hydrostatic mode-4 internal tide. For comparison the top two panels also show the SAR observations (black dots along the black solid line). (**c**) Velocities from the modal decomposition for mode-4 (in black) and modes-1, 2, 3 and 5 (in grey), preceding the SAR observation shown in Fig. 2. (**d**) Same as previous panel for the alternative bathymetry shown in top inset and in Fig. 5a.

Therefore, we first seek to investigate if the consistency between model and SAR seen in Fig. 3 for a given time frame holds throughout the time of simulations. This is done in Fig. 4a which shows a Hovmöller diagram using the modelled horizontal velocities (*U*) for the SAR observational period at a depth of 105 m (i.e. representative of IW propagation along the thermocline). It is clear that there is in fact a long eastward wave propagation originating approximately from the easternmost sill seen in Fig. 1 (hereafter East Sill, see also inset in Fig. 4a). Assuming the SAR observations in Fig. 2 (black dots in Fig. 4a) are generated in consecutive semidiurnal tidal cycles, the observed short-scale mode-2 solitary-like waves appear to align fairly well over time with the long wave propagation seen in the model Hovmöller diagram. The short mode-2 solitary-like waves, however, are hard to identify since their velocity (*U*) structures spread vertically over the bulk of the thermocline, and tend to fade out against other tidal beams and the longer vertical modes (see also Fig. 3).

To isolate the dominant vertical structure seen in the long wave propagation in Fig. 4a, we may separate the modelled velocity field in its vertical modes and investigate the existence of a long mode-4 internal tide. Using again a modal decomposition, the isolated contribution for mode-4 is shown in Fig. 4b. This figure shows that there is in fact a considerable amount of energy in IWs with a mode-4 vertical structure originating at semidiurnal tidal frequencies somewhere in the vicinities of the East Sill (i.e. mode-4 internal tides).

Furthermore, the observations in the SAR are seen to propagate with very similar speeds to those in Fig. 4b corresponding to a mode-4 internal tide, reinforcing the possibility of a resonant interaction between them. As usual a slight increase in propagation velocities is seen in the SAR observations when compared with the mode-4 internal tides (i.e. their slopes are slight different in Fig. 4b). Similarly to Fig. 3b, we recall this is usually attributed to the nonlinear contribution from the solitary-like waves.

The possibility of a resonant interaction was investigated by Magalhaes and da Silva^12^, mostly since mode-2 phase speeds in the SAR (around 0.6 to 0.7 m/s for depths greater than 1000 m) are very close to those predicted for a mode-4 internal tide with length-scales matching the observed inter-packet distances (see their Fig. 5). This larger mode-4 wave, however, cannot be verified by means of remote sensing. We therefore believe the simulations presented here build on their previous results – confirming the IW field has indeed a strong mode-4 internal tide component – and add to the possibility of a resonant coupling along the Ten-Degree Channel of the Andaman Sea.

**Figure 5:**
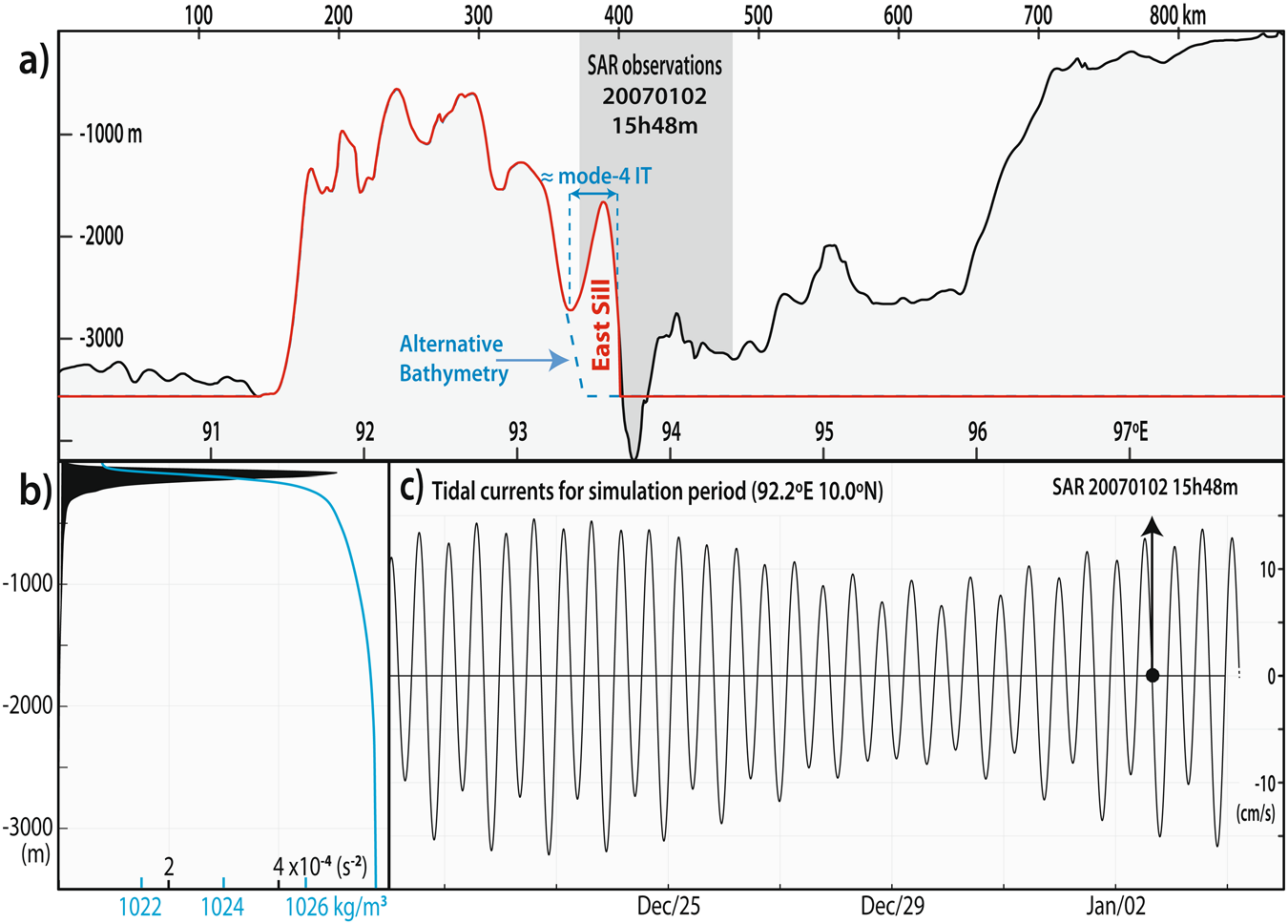
(**a**) Bathymetry along the model section (in black) and a simplified version used to run the numerical simulations (in red). The area corresponding to SAR observations shown in Fig. 2 is highlighted in grey. The alternative bathymetry used to simulate the effects of excluding the East Sill is shown in a light blue dashed line. (**b**) Background stratification used in the model (density in blue and Brunt-Väisälä frequency squared in black). (**c**) Zonal tidal currents at the top of the ridge.

The modal decomposition may be used to quantify contributions from other vertical modes as well. This is shown in Fig. 4c which compares mode-4 (in black) with the overall contribution from modes-1, 2, 3 and 5 (in grey) near WP1 (see Fig. 4). Typically, contributions from mode-1 alone would be expected to dominate in the Andaman Sea. This is not the case, however, along the Ten-Degree Channel which is consistent with the SAR where no mode-1 ISWs are observed as well. Furthermore, mode-4 clearly dominates over the other modes (note contributions decrease rapidly beyond mode-5), meaning the IW energy in the model leaving the forcing bathymetry is done mostly via a mode-4 internal tide.

Mode-4 internal tides are not commonly observed in the ocean, since lower modes usually dominate in most cases^26,30^. Therefore, it is interesting to note that the forcing bathymetry in this particular case appears to be a quasi-sinusoidal feature (labelled East Sill in Figs. 1, 4 and 5), whose characteristic length is around 30 km (considered at a depth of around 2500 m), and thus very close to the inter-packet distances in the SAR and the modelled wavelength for a mode-4 internal tide. To investigate this topographic feature, we used a synthetic bathymetry in the simulations, in which the East Sill is removed (see dashed blue line in Figs. 4a and 5), to simulate the effects thereof in the IW field. A similar plot to that in Fig. 4c is shown in Fig. 4d for this alternative bathymetry, and it shows that the resulting IW field is very distinct. Unlike Fig. 4c, the mode-4 contribution decreases considerably and the other modes dominate instead. In fact, the simulations in this case loose the mode-4 structure as well as the higher frequency surface signatures. Altogether, this means that in the simulations the East Sill plays an important role in the long-lived mode-2 solitary-like features observed along the Ten-Degree Channel of the Andaman Sea. Note also that, this bathymetry feature (i.e. the East Sill) runs approximately along the meridional extent of the observations (see Fig. 1) and hence is consistent with the SAR^12^.

We also note in passing, that background shear is known to add mixing into mode-2 solitary-like waves, meaning that the lack thereof could explain their longevity in the Andaman Sea^18^. In the Ten Degree channel, mode-2 solitary-like waves are observed most of the year, meaning they are likely to experience a wide range of background conditions (including shear) and still persist for at least 2 semidiurnal tidal periods (as observed in the SAR). Also, shear profiles are naturally introduced as the mode-4 internal tide propagates eastwards (see Fig. 3b). This means energy losses and leakage into other shear-induced waves is expected in the Andaman Sea (as explained in Zhang *et al*.^18^). However, this would only add to the case of a resonant coupling as an additional source to explain the waves’ extended longevity, and we therefore do not pursue a baroclinic steady flow in our simulations (especially since results agree well with the SAR ground truth).

Finally, removing or changing the steady current used in the simulations (within a reasonable extent) does not seem to affect the overall outcomes of the experiments (see also the Methods section). However, changing it shifts the positions of the modelled waves relative to those seen from the SAR, whereas the best agreement is for a −0.1 m/s (i.e. westwards at the top of the ridge) which is in reasonable agreement with currents observed and simulated along the Ten-Degree Channel of the Andaman Sea (see e.g. Fig. 11 in Chatterjee *et al*.^31^). Similarly, model runs along other sections farther north or south (within the range of the observations seen in Fig. 2) do not change the overall outcomes of the simulations as well.

### Summary

A high-resolution non-hydrostatic and fully nonlinear setup is used in the MITgcm to simulate the internal wave field along the Ten-Degree Channel of the Andaman Sea. Previous studies^12,13^ revealed the propagation of unusually long-lived mode-2 solitary-like waves along this particular stretch, whereas the usual large-scale mode-1 ISWs were never observed in satellite imagery. The model results are consistent with these observations since they too feature high-frequency mode-2 oscillations propagating along the pycnocline instead of the usual mode-1 solitary waves. While a proxy for their sea surface signatures in the model appears roughly in agreement with the SAR, their inter-packet distances compare well with a mode-4 internal tide vertical structure along the water column.

According to the model results, this larger mode-4 internal tide (whose surface signatures are beyond the imaging capabilities of the SAR) is likely to be generated as tidal flow oscillates over the easternmost sill of the Andaman and Nicobar Islands Ridge, which then propagates eastwards with phase velocities close to those estimated from SAR for the higher-frequency mode-2 solitary-like waves. Therefore, the stage is set for a resonance coupling between these two sets of waves, which would explain the extended longevity that is consistently seen in SAR for these frequently observed mode-2 solitary-like waves.

## Methods

### Model setup

Numerical simulations are performed with the Massachusetts Institute of Technology Global Circulation Model (MITgcm^32^) in a fully nonlinear and non-hydrostatic two-dimensional configuration with realistic bathymetry, stratification and tidal currents – all of which corresponding to the SAR observation in Fig. 2. The model setup follows from that used in Buijsman *et al*.^33^ for the Luzon Strait and in da Silva *et al*.^6^ in for Mascarene Ridge (see their section 2.2. for a detailed description), whereas in this case reduced grid spacing and time steps were used to model the short-scale mode-2 solitary-like waves in the Andaman Sea. The model stretches in the horizontal for 1800 km with 7000 grid cells. A higher resolution domain was set along the main ridge and the waves’ propagation path as observed in the SAR (roughly between 91.5 and 95 °E, see Fig. 5). In the centre part of the domain the grid cells are 75 m and they increase linearly to 9.7 km towards the boundaries. The increased resolution is needed to resolve the IW features anticipated in the SAR, which range from the shorter mode-2 solitary-like waves to the larger internal tides – a time step of 2 seconds ensured a proper Courant–Friedrichs–Lewy condition and stable model runs. The horizontal grid size also ensures that numerical dispersion is smaller than physical dispersion, allowing for proper non-hydrostatic effects^34,35^. Note that, in this case thermocline depths yield leptic ratios around 0.7. In the vertical the model includes 200 layers, with a grid spacing of 5 m in the top 600 m, which is stretched smoothly to 45 m towards the bottom. The model uses realistic topography with a 1 minute resolution^36^. The section used in the simulations is chosen to run along the waves direction of propagation (see Fig. 1), and extends along the Ten-Degree Channel for 900 km to the west and east of the waves’ forcing bathymetry (see Fig. 5a). Away from the ridge the model depth is set to a constant 3600 m.

Setting up proper background stratification is also important, especially since density is known to change both the modal content in the water column^30^, as well as propagation dynamics for mode-2 solitary-waves^17^. In the case of the Ten-Degree Channel, stratification is mostly like in other tropical seas where strong thermoclines are found below a mixed layer (i.e. no seasonal changes as in mid-latitudes). Therefore, a representative density stratification was obtained from a climatological mean for January (according to the SAR in Fig. 2 and shown in Fig. 5b, available at www.esrl.noaa.gov) – which is assumed constant along the horizontal in the model. We note in passing that, this stratification is similar to that found in Yang *et al*.^17^, which was found to be strongly correlated with optimum conditions for mode-2 ISWs, and it does not change significantly throughout the simulation period – regardless for instance of smaller effects introduce by tidal beams or the mode-4 internal tide. Moreover, a sensitivity analysis revealed no significant changes in the outcomes when changing stratification moderately within typical profiles in this study region.

The tidal forcing used in the model is shown in Fig. 5c and was computed using the Oregon Tidal Inversion Software^37^ as being representative of tidal currents at the top of the ridge. The simulations begin after 20 December 2006 and the model runs last for 17 days. This means the SAR observation in Fig. 2 is within the last days of the simulation and allows meaningful comparisons between the model and the realistic observations. To ensure realistic forcing, a sufficient amount of tidal constituents is used to reconstruct realistic tides (M_2_, S_2_, N_2_, K_1_, K_2_, O_1_, P_1_ and Q_1_), and a body force term is used to generate internal tides^38^ and added to the model as in da Silva *et al*.^6^.

Finally, we note that in the Andaman Sea near-surface circulation is in the clockwise direction between July and March, meaning a mean flow is set from the Andaman Sea into the Bay of Bengal along the Ten-Degree Channel^31^. This needs to be considered since steady currents have been shown to affect ISW dynamics in other known hotspots^6^. Therefore, asymmetrical tidal flow was used in the simulations, whereas the best agreement with the SAR observations was found for a westward flow of about 0.1 m/s at the top of the ridge, which we note is in fair agreement with nominal values for this particular stretch of the Andaman Sea^31^. We also recall that, even though recent studies have shown that vertical shear is important in the dynamics of mode-2 solitary-like waves^18^, we do not pursue a baroclinic steady flow in our simulations. Note that our barotropic approximation already covers the essence of the waves’ dynamics, especially since results agree well with the SAR ground truth.

### Modal decomposition

In this study a modal decomposition was used, which solves the Taylor-Goldstein equation governing linear IW motion in a stratified vertically sheared mean flow with appropriate boundary conditions (as in da Silva *et al*.^6^), and computes the eigenfunctions (i.e. the vertical modes) and eigenvalues (i.e. the corresponding linear phase speeds for each mode) according to Eq. (1) . In this formulation, $\phi$ is the modal structure, *k* is the wavenumber representing the non-hydrostatic term, *N* is the Brunt-Väisälä frequency, *U* is the flow velocity along the waves’ direction of propagation, *c* is a characteristic phase speed, and *H* is the bottom depth.

1 $$\frac{{d}^{2}\phi }{d{z}^{2}}+\left[\frac{N{(z)}^{2}}{{(U-c)}^{2}},-,\frac{\frac{{d}^{2}U}{d{z}^{2}}}{U-c},-,{k}^{2}\right]\phi =0,\,\phi (0)=\phi (-H)=0$$

When applying this technique to the model data, the eigenfunctions from Eq. (1) are fitted (i.e. projected) onto the model velocities to extract modal amplitudes for each mode^39,40^. Therefore, the modelled velocity field may be reconstructed simply by adding the individual contributions for each mode – which in turn are computed by multiplying the modal amplitudes by the corresponding eigenfunctions. This means that each modal constituent in the modelled velocity field may be analysed separately. This is especially important here since our observations are close to forcing bathymetry, and hence to internal tidal beams (see e.g. the tidal beam in Fig. 3a) which result from the superposition of several vertical modes. We recall that only linear modes are solved in a modal decomposition, and hence no contributions from nonlinear waves are accounted for. Nonetheless, a modal decomposition using the first twenty vertical modes was used in this study, which efficiently reproduced the model results (e.g. differences are less than 5% in the case of Fig. 4a).

## Data Availability

SAR imagery is available from https://esar-ds.eo.esa.int/oads/access/. Tidal currents are available from http://people.oregonstate.edu/~erofeevs/otis.html, and the bathymetry dataset follows from Smith and Sandwell (1997, available at https://topex.ucsd.edu/marine_topo/). The numerical modelling data used in this manuscript are available on request.
